# Effects of 6-week sprint interval training compared to traditional training on the running performance of distance runners: a randomized controlled trail

**DOI:** 10.3389/fphys.2025.1536287

**Published:** 2025-02-06

**Authors:** Kai Jin, Mengbiao Cai, Yongqian Zhang, Bin Wu, Yi Yang

**Affiliations:** ^1^ Department of Physical Education, Southeast University, Nanjing, China; ^2^ Department of Physical Education, Nanjing University of Aeronautics and Astronautics, Nanjing, China; ^3^ Department of Science and Graduate Studies, Tianjin University of Sport, Tianjin, China; ^4^ Department of Physical Education, Nanjing City Vocational College, Nanjing, China; ^5^ College of Physical Education, Hengxing University, Qingdao, China

**Keywords:** sprint interval training, VO2max, running performance, distance runners, endurance

## Abstract

**Introduction:**

This study aimed to compare the effects of sprint interval training *versus* traditional training on running performance in well-trained male distance runners.

**Methods:**

Twenty male distance runners (VO2: 67.4 ± 4.5 mL/kg/min, personal best time for the 5000 m: 14′38″47 ± 00′23″46) were recruited and randomly assigned to either the intervention training (IT) group, which performed sprint interval training, or the control training (CT) group, which engaged in traditional long-distance training. Both groups completed their respective training regimens twice a week for 6 weeks. Measurements for VO2max, O2 cost, time to exhaustion (TTE), and running times for 100, 400, and 3000 m were taken before and after the intervention.

**Results:**

The results indicated that the IT group showed significant improvements in TTE and running performance across 100, 400, and 3000 m (all P < 0.01), while the CT group only demonstrated improvements in 400 m time (P < 0.01). The IT group exhibited superior 3000 m performance compared to the CT group (P < 0.01). Analysis of effect sizes revealed small to moderate improvements in physiological and performance measures for the IT group, with VO2max showing a small effect size of 0.43, O2 cost a moderate effect size of 0.65, and TTE a moderate effect size of 0.77.

**Conclusion:**

These findings suggest that sprint interval training may offer superior benefits for enhancing running performance of well-trained male distance runners, particularly in time to exhaustion and middle-to long-distance events, compared to traditional longdistance training.

## 1 Introduction

Interval training is a structured form of exercise that alternates between high-intensity bursts of activity and periods of rest or lower intensity. This training method is designed to enhance both aerobic and anaerobic fitness by pushing the body to work at maximum effort during the intense phases, followed by recovery periods that allow for partial recuperation (Gist et al., 2014; Gibala, 2021). Research has demonstrated that interval training can induce physiological adaptations comparable to those achieved through traditional endurance or strength training, effectively combining the benefits of both (Burgomaster et al., 2008; MacInnis and Gibala, 2017; Boullosa et al., 2022). For example, a study by McRae et al. compared the effects of whole-body interval training with traditional endurance training in active females, revealing that interval training offered significant additional benefits in enhancing aerobic fitness and muscular endurance (McRae et al., 2012). Interval training is designed to surpass the adaptations achieved through traditional long-duration, steady-intensity training by incorporating short bursts of high-intensity effort followed by recovery periods. These high-intensity intervals push the body to work at or near maximum effort, stimulating both aerobic and anaerobic systems more effectively than continuous steady-state exercise. Recent studies have demonstrated that this training method not only improves cardiovascular endurance and aerobic capacity (Milanović et al., 2015), but also enhances muscle oxidative capacity and metabolic flexibility, leading to greater overall performance (Hood et al., 2011; Callahan et al., 2021). Furthermore, interval training allows for greater time efficiency while yielding comparable or superior benefits to longer, moderate-intensity training protocols (Billat et al., 2000; Gillen and Gibala, 2014). This is particularly relevant for elite endurance athletes, who typically engage in extensive training and therefore need to effectively manage their total training duration while enhancing training efficiency. Research indicates that interval training has become a central component of training for athletes in endurance sports such as middle-distance running, long-distance swimming, and cycling (Hellard et al., 2019; Leo et al., 2020; Casado et al., 2022). Thus, given its proven benefits, interval training, particularly sprint interval training (SIT), has become an essential component of training for endurance athletes, as it offers a time-efficient alternative to traditional endurance training while enhancing both aerobic and muscular performance.

Sprint interval training (SIT) is a specialized form of high-intensity exercise that involves short bursts of maximum effort followed by longer rest periods. This training method is characterized by short bursts of activity and extended recovery (Mølmen and Rønnestad, 2024; Usher and Babraj, 2024). The sprints are repeated multiple times and the recovery period is interspersed between every two sprints. SIT offers the same benefits as other interval training methods, enhancing aerobic capacity and muscular performance when implemented in a well-structured training protocol (Skovgaard et al., 2018). For instance, a study by Gibala et al. investigated the effects of low-volume SIT compared to high-volume endurance training in active males, revealing similar improvements in muscle oxidative capacity, muscle buffering capacity, and muscle glycogen content for both training methods (Gibala et al., 2006). SIT is particularly beneficial for endurance athletes, and developing targeted SIT protocols is essential for effective training management among elite athletes. Research demonstrates that multiple all-out sprints lasting no more than 40 s, with recovery periods 5 times longer than the sprint duration, can significantly improve the performance of endurance athletes (Bangsbo, 2015).

Additionally, training intensity—specifically, all-out sprints of varying durations—emerges as a key loading parameter in SIT (Boullosa et al., 2022). The most commonly used SIT protocol involves performing 4 to 6 repetitions of 30-s all-out sprints, with numerous studies confirming the positive effects of this approach (Vollaard and Metcalfe, 2017; Vollaard et al., 2017). However, despite the proven value of SIT, existing research is still insufficient. There is relatively little research on other parameters of the SIT protocol, such as repetition rate, training frequency, and training period (Vollaard and Metcalfe, 2017). Meanwhile, most SIT studies have been conducted in controlled laboratory environments, typically using equipment such as power bicycles or treadmills, which are both expensive and time-consuming, and not conducive to simultaneous training of multiple subjects (Astorino et al., 2012; Jakeman et al., 2012; Willoughby et al., 2016). As a result, there is a notable lack of field-based studies that assess the efficacy of SIT in real-world settings, with only a few exceptions (Koral et al., 2018), highlighting the need for further research in this area to better understand the practical implications of SIT for athletes in natural training environments.

Therefore, the primary aim of this study is to compare the effects of a carefully designed SIT protocol with traditional endurance training methods on the aerobic capacity and athletic performance of well-trained long-distance runners. This protocol conducts onsite and includes parameters such as sprint repetition frequency, training frequency, training cycle, sprint duration, and interval time. The hypothesis of this study posits that 12 sessions of maximum sprint training—each consisting of 10 sets of 30 s with 3.5 min of rest in between—over a period of 6 weeks, can significantly improve the VO2max, O2 cost (an index of running economy), time to exhaustion (TTE), and timed running performance in the 100, 400, and 3,000 m for long-distance runners.

## 2 Methods

### 2.1 Subjects

Twenty well-trained male distance runners were recruited for this study, with a mean VO2 max of 67.4 ± 4.5 mL/kg/min and an official personal best time for the 5000 m of 14′38″47 ± 00′23″46. The subjects were randomly divided into two groups using computer randomization: the intervention training group (IT group, n = 10, age: 19 ± 1 year, height: 173.3 ± 5.5 cm, weight: 59.5 ± 4.4 kg) and the control training group (CT group, n = 10, age: 20 ± 1 year, height: 175.8 ± 6.4 cm, weight: 58.6 ± 6.3 kg). Before the experiment commenced, the purpose of the study, its procedures, and poten-tial risks were thoroughly explained to all subjects, and written informed consent was obtained. The study was performed in accordance with the Declaration of Helsinki and was approved by the Ethics Committee of Sports Science Experiment (TJUS2024-046).

### 2.2 Research design

A randomized controlled trail (RCT) was conducted for this study. Subjects in both the IT group and the CT group performed high-intensity training twice per week for 6 weeks (12 sessions in total). The IT group engaged in sprint interval training for their high-intensity sessions, while the CT group participated in traditional long-distance training. Before the intervention, All subjects followed identical training regiments. Both groups engaged in regular aerobic endurance training, consisting of moderate-intensity jogging for 40–60 min, 4 days a week. Additionally, both groups took 1 day off per week. On the remaining days of the week, both the IT group and the CT groupjogged for 40–60 min on 4 days and took 1 day off. Before the tests, subjects were instructed to rest for at least 24 h after their last training session to minimize fatigue and ensure consistent test results. Post-intervention testing was performed 48 h following the final training session to allow for adequate recovery and avoid residual fatigue from the training sessions. Subjects were provided with general dietary guidelines before the test sessions to ensure that nutritional factors did not influence the results. They were instructed to consume a balanced meal consisting of complex carbohydrates, lean protein, and vegetables at least 3–4 h before each test session. Additionally, participants were advised to avoid heavy meals or caffeine intake within 2 h of the test. Before and after the 6-week training periods, VO2max, O2 cost, and TTE during submaximal running were assessed using a laboratory treadmill test. Additionally, sprinting times for 100 and 400 m, as well as the 3,000 m running time, were measured through field tests. The tests were scheduled before the intervention (baseline tests) and after the intervention (post-test) at specific intervals. Baseline tests were conducted within 1–2 days of the beginning of the intervention. Post-intervention tests were conducted 48 h after the final training session. For the field tests, the 100m and 400 m sprint tests were conducted on 1 day, and the 3000 m running test was conducted on a separate day within 5 days following the last training session.

### 2.3 Training protocols

The IT protocol was designed based on the research of Skovgaard et al. (Skovgaard et al., 2018). The protocol consisted of 10 sets of 30-s maximal sprint runs, with a 3.5-min recovery period between sets. During the recovery periods, subjects’ heart rates were maintained below 70% of their maximum. The starting workload for the first set was determined based on each subject’s average workload from their 100-m performance. Within each training session, the intensity was gradually reduced from interval to interval, as sustaining the high intensity of a 30-s maximal sprint is not feasible for 10 consecutive bouts. The average interval intensity during a training session was approximately 175% of the maximal aerobic speed (MAS). During all intervals, each subject received one-on-one follow-up and verbal encouragement to ensure maximum intensity during each sprint. At the end of each session, a 3-min cooldown was added, with an intensity corresponding to ≤70% of HRmax, resulting in a total duration of 50 min. Additionally, the CT protocol included various traditional endurance training programs over 12 sessions, was based on established endurance training methods that focus on improving aerobic capacity through sustained, moderate-intensity exercise, featuring 10-km running, 2 repetitions of 3000-m running, three repetitions of 2000-m running, 4 repetitions of 1500-m running, five repetitions of 1000-m running, and 12 repetitions of 400-m running (Campos et al., 2022). This protocol aimed to simulate a more traditional long-distance running approach commonly used by endurance athletes. The intensity of these sessions was prescribed based on a heart rate zone corresponding to 70%–85% of each subject’s maximum heart rate, which is typical for endurance training aimed at improving aerobic capacity. Each of these program was conducted twice.

### 2.4 Testing procedure and measures

#### 2.4.1 Treadmill running tests

VO2max, O2 cost, and TTE were measured using step and graded exercise tests conducted on a treadmill. The tests began with a 5-min warm-up at a speed of 200 m/min. Following the warm-up, subjects engaged in the step exercise test, which consisted of running at five different step speeds (230, 250, 270, 290, and 310 m/min) on a flat treadmill (0% slope). Each step lasted 3 min, followed by a 1-min rest period between steps. This approach has been shown to effectively reflect the running performance of long-distance runners (Tanji et al., 2017). After completing the step exercise test, subjects underwent a 3-min rest period before performing the graded exercise test. This test initiated at a speed of 290 m/min, with an increase of 10 m/min each minute (290, 300, 310, 320, 330, 340, 350, 360, 370 m/min, etc.), allowing subjects to reach their speed until exhaustion on the flat treadmill. During both exercise tests, a respiratory gas analyzer (AE-310S; Minato Medical Science), calibrated with a standard injector and precision reference gases prior to each test, was used to measure the expired levels of O2 and CO2 for each subject. VO2max was defined as the highest oxygen intake of the two tests. O2 cost was calculated as the average oxygen uptake during the final 60 s of steps 4 (290 m/min) and 5 (310 m/min) of the step exercise test. TTE was recorded as the duration of the graded exercise test. These treadmill running tests were conducted both before and after the intervention, with post-intervention tests per-formed within 2 days following the final session of the intervention.

#### 2.4.2 Field track running tests

The 100-m and 400-m running tests were conducted on an all-weather track utilizing a wireless phototube timing system (Brower TCi Timing Systems, HaB International Ltd.). The times were recorded between two timing gates, with a starting cue provided by the tester. During the tests, subjects were required to wear spikes and complete two 100-m sprints from a standing position, following a warm-up. A 5-min rest period was taken between the two sprints. The shorter of the two times was used for analyses. Subsequently, after a 10-min rest period, subjects proceeded to perform a 400-m sprint test from a standing start. To minimize the influence of external factors such as wind speed, we implemented a rigorous testing protocol that ensured all 20 subjects were tested within a 3-h timeframe. Additionally, the 3000-m timed run was conducted on the same all-weather track. To prevent subjects from adjusting their pace during the test, each subject completed the run individually. A tester used a stopwatch to record lap times at 1,000 and 2000 m, as well as the final completion time. All tests were conducted both before and after the intervention, with the post-intervention assessments for the 100-m and 400-m sprints taking place on the third day following the final session, and the post-intervention 3000-m run conducted within 5 days thereafter.

### 2.5 Statistical analyses

The test data were analyzed using IBM SPSS statistical software package (version 24.0). Descriptive statistics are presented as means ± standard deviation (M±SD). The normality of all variables was confirmed using the Shapiro-Wilk test. Inter-group dif-ferences between the IT group and CT group across various indicators (VO2max, O2 cost, TTE, 100 m-time, 400 m-time, and 3000 m-time) were evaluated using independent t-tests, while intra-group differences were assessed using paired t-tests, with Bonferroni adjustment applied for multiple comparisons. Cohen’s d was used to assess the effect size (ES) according to the following thresholds: <0.2 as trivial, 0.2–0.6 as small, 0.6–1.2 as moderate, 1.2–2.0 as large, and >2.0 as very large (Hopkins et al., 2009). The level of significance was set at P < 0.05 for all tests.

## 3 Results

The M ± SDs and performance changes are presented in Tables 1, 2. The Shapiro-Wilk tests confirmed that all data followed a normal distribution. Intra-group comparisons revealed that the IT group demonstrated significant improvements in TTE and performance for the 100m, 400m, 3000 m running following training, all with statistical significance (P < 0.01). In contrast, VO2max and O2 cost did not exhibit sta-tistically significant changes in the IT group, with P-values of 0.055 and 0.077, respectively. Within the CT group, only the 400 m time showed a significant change com-pared to pre-training (P < 0.01), while no statistically significant differences were ob-served found in the other variables (Table 1).

**TABLE 1:** Intra-group comparisons of variables before (pre-test) and after (post-test) training program.

Variables	IT group (n = 10)				CT group (n = 10)			
	Pre	Post	Mean changes	P-values	Pre	Post	Mean changes	P-values
VO_2_ max	4.00 ± 0.31	4.17 ± 0.34	0.17 ± 0.23	0.055	4.03 ± 0.23	4.04 ± 0.25	0.01 ± 0.03	0.366
O_2_ cost	3.34 ± 0.09	3.22 ± 0.15	−0.11 ± 0.18	0.077	3.32 ± 0.10	3.30 ± 0.06	−0.02 ± 0.05	0.365
TTE	8.30 ± 0.22	8.42 ± 0.18**	0.12 ± 0.08	<0.01	8.27 ± 0.23	8.26 ± 0.23	−0.01 ± 0.02	0.423
100 m-time	13.49 ± 0.75	13.29 ± 0.68**	−0.20 ± 0.10	<0.01	13.22 ± 0.67	13.23 ± 0.69	0.01 ± 0.02	0.471
400 m-time	56.10 ± 2.12	55.44 ± 2.14**	−0.67 ± 0.50	<0.01	55.51 ± 3.28	55.23 ± 3.21**	−0.28 ± 0.19	<0.01
3000 m-time	527.48 ± 4.44	522.61 ± 3.52**	−4.86 ± 1.36	<0.01	528.23 ± 4.12	527.93 ± 3.44	−0.30 ± 1.12	0.414

*p < 0.05; **p < 0.01; VO2 max, maximal oxygen uptake; O2 cost, Oxygen Cost; TTE, time to exhaustion.

**TABLE 2:** Pairwise comparisons between groups of variables before (pre-test) and after (post-test) training program.

Variables	Pre-test			Post-test		
	Mean changes	P-values	Cohen’s d	Mean changes	P-values	Cohen’s d
VO_2_ max	−0.03 ± 0.12	0.803	0.11	0.13 ± 0.13	0.353	0.43
O_2_ cost	0.02 ± 0.04	0.619	0.23	−0.08 ± 0.05	0.162	0.65
TTE	0.03 ± 0.10	0.762	0.14	0.16 ± 0.09	0.102	0.77
100 m-time	0.27 ± 0.32	0.400	0.39	0.07 ± 0.31	0.834	0.09
400 m-time	0.59 ± 1.23	0.639	0.21	0.21 ± 1.22	0.867	0.08
3000 m-time	−0.75 ± 1.91	0.699	0.18	−5.31 ± 1.56**	<0.01	1.53

*p < 0.05; **p < 0.01; VO2 max, maximal oxygen uptake; O2 cost, Oxygen Cost; TTE, time to exhaustion.

Baseline comparisons revealed no significant differences in any variables between the IT and CT groups prior to the training program (Table 2). Post-training inter-group comparisons indicated that the IT group achieved a significant reduction in 3000 m time compared to the CT group (P < 0.01). However, no statistically significant differ-ences were found for VO2max, O2 cost, TTE, 100 m time, or 400 m time. Further analysis of ESs revealed that VO2max had a small ES (0.43), while O2 cost and TTE have moderate ESs (0.65 and 0.77, respectively). The 3000 m time had a large ES (1.53) (Table 2).

## 4 Discussion

This study demonstrated that SIT, consisting of 10 sets of 30-s maximum sprint runs with a 3.5-min recovery period, can significantly enhance the TTE of male long-distance runners, as well as their performance in the 100m, 400m, and 3000 m events. In comparison to CT, SIT shows a small effect size on VO2max, a moderate effect size on O2 cost and TTE, and a large effect size on 3000 m performance. These findings contribute to the expanding body of literature that supports SIT as an effective training modality for endurance athletes.

Our study used VO2max, O2 cost, and TTE to assess the athletic performance and endurance of long-distance runners. VO2max is widely regarded as the best indicator of cardiovascular endurance (Jansson and Kaijser, 1987; Millet et al., 2023), and O2 cost is used to evaluate exercise economy (Scheer et al., 2018). VO2max represents the maximal capacity of an individual’s cardiovascular system to transport and utilize oxygen during intense exercise. It is a crucial determinant of aerobic endurance, as higher VO2max values are associated with improved performance in endurance-based events, such as long-distance running (Broxterman et al., 2024). Lower O2 cost indicates greater exercise economy, reflecting the efficiency with which the body utilizes oxygen at a given intensity, thereby reducing energy expenditure and enhancing performance over prolonged periods (Jones et al., 2021). In this study, although no significant improvements in VO2max and O2 cost were observed with high-intensity SIT, which contradicts previous research (Buchheit and Laursen, 2013; Thurlow et al., 2024), the results with P-values close to 0.05 suggest a potential effect may exist. This lack of significant improvement could be attributed to several factors, including the short duration of the training program and the possibility that SIT might preferentially target other physiological adaptations, such as muscle oxidative capacity or neuromuscular function, which may not immediately impact VO2max and O2 cost. Additionally, the intergroup differences in this study revealed that the VO2max of the IT group was higher than that of the CT group, and the O2 cost was lower in the IT group, indicating an advantage in cardiovascular endurance and exercise economy for the IT group. This trend aligns with the notion that high-intensity interval training can enhance muscle oxidative capacity, which indirectly improves VO2max, although these changes may not be immediately observable in a short intervention (Wang and Wang, 2024). While the statistical differences did not achieve significance, the small to medium effect sizes underscore the need for further research. Studies comparing SIT to traditional training have shown conflicting results regarding VO2max and O2 cost. Some studies suggest that while SIT may lead to improvements in other performance markers such as TTE, it may not always produce the same magnitude of change in VO2max or O2 cost compared to traditional endurance training (Buchheit and Laursen, 2013). However, the current study’s small sample size and short intervention period may limit the detection of such changes, suggesting that longer training periods or larger sample sizes might be necessary for clearer insights. Future studies could consider increasing the sample size or extending the intervention duration to obtain more definitive results.

For TTE, it is a key metric in exercise physiology that measures an individual’s endurance capacity (Amann et al., 2008). TTE evaluates aerobic capacity, muscular endurance, and overall fitness; a longer TTE suggests better endurance performance (Chen et al., 2022; Sitko et al., 2022). In this study, the significant increase in TTE was observed, suggesting that SIT may effectively enhance both muscular endurance and aerobic capacity in long-distance runners. These findings are consistent with previous studies comparing SIT to traditional endurance training, which also found that SIT can significantly improve TTE, potentially by promoting both aerobic and anaerobic adaptations (Buchheit and Laursen, 2013). However, it is important to note that while SIT has demonstrated improvements in TTE, its effects may vary depending on the training parameters, such as work-to-rest ratios and the intensity of the sprints. For example, shorter recovery periods may yield more pronounced anaerobic adaptations, while longer recovery periods, as used in this study, may favor aerobic improvements (MacInnis and Gibala, 2017). The mechanism behind this improvement could be attributed to various physiological adaptations, including increased muscle oxidative capacity and enhanced neuromuscular function (Burgomaster et al., 2005; Little et al., 2010), both of which are essential for sustaining prolonged exercise efforts. This supports the view that SIT may enhance aerobic capacity through increased mitochondrial density and oxidative enzyme activity in the muscles, allowing athletes to sustain higher intensities for longer durations. Specifically, SIT can enhance the ability of muscles to produce more energy through aerobic pathways, allowing athletes to maintain high-intensity exercise for extended periods and effectively delay the onset of fatigue (Burgomaster et al., 2005; Gibala et al., 2006; Little et al., 2010; Gibala et al., 2012; MacInnis and Gibala, 2017; Gonzalez Rojas et al., 2024). Additionally, SIT can improve the coordination between the nervous system and muscles, increasing the efficiency with which the nervous system activates the muscles involved in running (Bertschinger et al., 2020), which helps athletes achieve better performance in endurance events.

Another critical outcome of this study is the significant enhancement in running performance across various distances. The improvements observed in the 100m and 400 m events indicate that SIT not only enhances anaerobic capacity but also positively influences speed and power output. For long-distance runners, speed and power output are essential components of overall performance, as they contribute to the ability to sustain fast paces over long periods and recover from surges in race intensity (Beattie et al., 2014). In particular, speed helps runners maintain competitive paces during race segments, while power output enables better acceleration and efficient use of energy, which are critical when running up hills or during sprint finishes (Bazyler et al., 2015). These results align with previous research high-lighting the benefits of high-intensity training for neuromuscular adaptations and sprint mechanics (Buchheit and Laursen, 2013). Specifically, these neuromuscular adaptations include increased muscle fiber recruitment and faster muscle contraction rates, which help athletes maintain more efficient movement patterns, reduce energy expenditure, and enhance sprint performance (Du and Sim, 2021; Van der Stede et al., 2024). By improving neuromuscular coordination, SIT helps athletes produce greater force with less effort, which improves running economy and reduces fatigue. The sprint mechanics associated with SIT suggest that it improves the anaerobic energy systems critical for 100m and 400 m sprints. By en-hancing the muscles’ ability to generate energy anaerobically, SIT helps athletes manage lactate accumulation more effectively and delay the onset of fatigue (Fiorenza et al., 2019; Thom et al., 2020).

In this study, the significant improvements in the 3000 m event indicate that SIT can also enhance aerobic performance. This is particularly significant, as the 3000 m race requires a balance between both aerobic and anaerobic energy systems. These results align with several studies that have compared the effects of SIT *versus* traditional endurance training on long-distance performance. Research has shown that, while traditional endurance training primarily enhances aerobic capacity and improves performance in sustained, moderate-intensity events, SIT may offer additional benefits by improving both anaerobic capacity and overall race performance, especially in middle- and long-distance events (Buchheit and Laursen, 2013). The physiological adaptations associated with SIT, such as enhanced muscle oxidative capacity and improved lactate threshold, likely play a crucial role in maintaining higher intensities over extended periods (MacInnis and Gibala, 2017). Additionally, SIT’s ability to improve neuromuscular function and running economy has been suggested to allow athletes to maintain faster paces and recover more effectively from surges in pace, which is crucial for 3000 m races that require both aerobic endurance and intermittent anaerobic efforts (Laursen and Jenkins, 2002). This interval format ensures that athletes can sustain anaerobic efforts during the sprints while still benefiting from the aerobic adaptations that occur during the recovery phase between bouts of high-intensity exercise (Jones and Carter, 2000). This recovery period is also sufficient to prevent complete depletion of phosphocreatine stores, allowing athletes to perform repeated high-intensity efforts with reduced fatigue compared to continuous endurance training (Bishop et al., 2011).

This study has several limitations. First, the sample size was relatively small, consisting of only 20 participants, which may increase the influence of individual variations on the results. To enhance the robustness and generalizability of the findings, future research should aim to include a larger sample. Additionally, the study population was limited in terms of gender and age range, as all participants were male and within a specific age group (e.g., 18–30 years). This homogeneity may reduce the generalizability of the results to other populations, such as female athletes or older individuals. Future studies should consider including a more diverse sample, encompassing both genders and a wider age range, to determine if the effects of SIT on endurance performance differ across these variables. Second, the duration of the intervention was limited to 6 weeks. Although this timeframe is adequate for observing short-term effects, it is insufficient for evaluating the long-term impact of SIT on ath-letic performance. Furthermore, the level of trainability of participants, which may vary depending on their training history and experience, was not considered. This variability could influence the responsiveness to SIT. In future studies, the training background of participants should be documented and analyzed to assess how different levels of baseline fitness might affect the outcomes of SIT. Future studies could benefit from extending the intervention period to better assess the enduring effects of the training regimen.

## 5 Conclusion

Our findings suggest that SIT is a time-efficient and effective alternative to traditional continuous training for long-distance runners. As athletes seek to optimize training while minimizing time commitment, SIT could be a practical addition to training programs. For coaches and athletes, it is recommended to incorporate two to three sessions of SIT per week, focusing on high-intensity sprints followed by adequate recovery, as a way to complement traditional endurance training. This approach can enhance both aerobic and anaerobic capacity while reducing the overall training time required. Future research should explore the long-term effects of SIT on performance and its underlying physiological mechanisms, as well as the optimal integra-tion of SIT with traditional endurance training. Additionally, it would be beneficial to investigate how SIT can be tailored to different athlete populations, such as those with varying fitness levels or training backgrounds, to maximize its impact. Overall, our results support the inclusion of SIT as a valuable tool for enhancing performance in male long-distance runners.

## Data Availability

The raw data supporting the conclusions of this article will be made available by the authors, without undue reservation.
